# Effects of A 4-Week Aerobic Exercise on Lipid Profile and Expression of *LXRα* in Rat Liver 

**DOI:** 10.22074/cellj.2016.4871

**Published:** 2016-12-21

**Authors:** Fatemeh Kazeminasab, Mohammad Marandi, Kamran Ghaedi, Fahimeh Esfarjani, Jamal Moshtaghian

**Affiliations:** 1 Department of Exercise Physiology, Faculty of Sport Sciences, University of Isfahan, Isfahan, Iran; 2 Department of Biology, Faculty of Sciences, University of Isfahan, Isfahan, Iran

**Keywords:** Treadmill Exercise, Liver X Receptor α, High-Density Lipoprotein Cholesterol, Rat

## Abstract

**Objective:**

Liver X receptors (LXRs) are ligand-activated transcription factors of the nuclear hormonal receptor superfamily which modulate the expression of genes involved in cholesterol homeostasis. Hence, further unraveling of the molecular function of this gene may be helpful in preventing cardiovascular diseases.

**Materials and Methods:**

This experimental intervention study included twelve adult Wistar male rats (12-14 weeks old, 200-220 g) which were divided into the control (n=6) and training (n=6) groups. The training group received exercise on a motor-driven treadmill at 28 meters/minute (0% grade) for 60 minutes a day, 5 days a week for 4 weeks. Rats were sacrificed 24 hours after the last session of exercise. A portion of the liver was excised, immediately washed in ice-cold saline and frozen in liquid nitrogen for extraction of total RNA. Plasma was collected for high-density lipoprotein cholesterol (HDL-C), low-density lipoprotein cholesterol (LDL-C), total cholesterol (TC) and triglycerides (TG) measurements. All variables were compared by independent t test.

**Results:**

A significant increase in *LXRα* transcript level was observed in trained rats (P<0.01). Plasma HDL-C concentration was also significantly higher (P<0.01) in trained rats. There was a significant decrease in the concentrations of LDL-C (P<0.01) and TC (P<0.02), and the ratios of TC/HDL-C (P<0.001) and LDL/HDL-C (P<0.002) in trained rats. However, the TG concentration was unchanged (P>0.05).

**Conclusion:**

We found that endurance training induces significant elevation in *LXRα* gene expression and plasma HDL-C concentration resulting in depletion of the cellular cholesterol. Therefore, it seems that a contributor to the positive effects of exercise in cardiovascular disease prevention is through the expression of LXRα, which is a key step in reverse cholesterol transport.

## Introduction

Coronary artery disease (CAD) is among the greatest causes of morbidity and mortality in most countries and is strongly correlated with increase in plasma total cholesterol (TC), low-density lipoprotein cholesterol (LDL-C), and high-density lipoprotein cholesterol (HDL-C) concentrations (1,6). Reverse cholesterol transport (RCT) is a complex process resulting in the net movement of cholesterol. In this process, the formation and remodeling of HDL-C in plasma requires several factors. The ATP-binding cassette transporter A1 (ABCA1), lecithin cholesterol acyltransferase (LCAT), lipoprotein lipase (LPL), hepatic lipase (HL), cholesteryl ester transport protein (CETP) and phospholipid transport protein (PLTP) are among these factors (2,7,8). 

Liver exerts its lipid-regulating function through numerous receptors. One of these receptors which play a key role in cholesterol metabolism is the liver X receptor (LXR). LXRs are ligand-activated transcription factors of the nuclear receptor superfamily that are thought to play a role in the regulation of liver lipid metabolism (9). The two LXR subtypes α and β form heterodimers with retinoid X receptor upon activation and bind to the LXR response element found in the promoter region of the target genes (9,12). Both are involved in regulating the expression of genes involved in cholesterol homeostasis (*ABCA1, ABCG1*) (10,13,14). 

LXRs stimulate the reverse cholesterol transport through increasing the expression of *ABCA1* gene and enhancing the availability of extracellular cholesterol acceptors including apolipoprotein E (15,16). Previous study has have shown that the natural and synthetic agonists of LXRs cause an increase in the expression of *ABCA1* and excretion of cholesterol from the cells. Hence, they may be potential therapeutic agents for preventing arthrosclerosis (17). 

To the best of our knowledge, effects of regular exercise on blood-lipid and lipoprotein profiles have been well established. Also, exercise has been shown to improve the capacity of cardiovascular function and enhance the reverse cholesterol transport process, resulting in up-regulation of plasma HDL (6,7,18,19). Butcher et al. (20) reported that low intensity exercises (1000-step walking) in 3 sessions per week caused an increase in *LXRα* gene expression in human leukocytes. However, the knowledge about the effect of exercise on the expression of *LXRα* in liver is not established. We thus aimed to investigate the expression of *LXRα* in rat liver along with HDL-C, LDL-C, TG and TC concentrations after 4 weeks of treadmill exercise training. 

## Materials and Methods

This animal-based experimental intervention study was approved by the Research Committee of the University of Isfahan (Office of Research Affairs) according to the policy of the Ethics Committee of University of Isfahan. 

### Animals

Twelve male Wistar rats with an estimated weight of 200-220 g were kept under normal light conditions (12 hours light dark cycle), temperature (23 ± 1˚C) and moisture of (50 ± 3%) in special cages. The rats were fed a pellet rodent diet ad libitum and had free access to water. The whole process was carried out by the same person. After two weeks of work in the laboratory to minimize the effect of human intervention, animals were randomly assigned to the control (n=6) and training (n=6) groups. 

### Exercise training protocol

The training program began with adapting rats with the apparatus for 7 days by placing them on a motor-driven treadmill (School of Medicine, Isfahan University of Medical Sciences). The training protocol started with the rats receiving exercise on the treadmill at 16 meters/minute for 15 minutes. One week after the initial stage, the time and speed of running was increased steadily to 60 minutes per day at 23 meters/minute. After this stage, the rats of the training group were put into a progressive exercise. They were put again on a treadmill to run for 60 minutes per day, 5 days a week. During the first week, the speed was set to 20 meters/minute, while for the second and third and fourth weeks it was adjusted to 25, 27 and 29 meters/minute respectively. The angle of inclination was 0˚ during the whole study period. This condition corresponded to a moderate intensity with about 65% of maximal oxygen consumption (21,22). 

### Liver biopsy and blood samples

Twenty-four hours after the last exercise session (fourth week), the rats were anesthetized intraperitoneally with a mixture of ketamine (30-50 mg/kg of body weight) and xylazine (3-5 mg/kg of body weight). After confirming unconsciousness by observing no foot reaction to a physical stimulant, 3 mL of the blood was taken from the right ventricle of each rat and immediately poured into a test tube. The blood samples were centrifuged for 15 minutes at 4000 rpm to separate the blood serum. The obtained sera were kept in a deep freezer (80˚C) for future measurements. 

After collecting the blood samples, the abdominal part of the rats were cut and a portion of the liver was excised and washed in ice-cold saline. The samples were then immediately frozen in liquid nitrogen for total RNA extraction. The frozen liver tissues were kept at -80˚C for further experiments. 

### Lipid and lipoprotein measurements

The concentration of TC, TG and HDL-C were measured in a calibrated biochemical analyzer (Hitachi 902 Automatic analyzer, Japan) in the following manner. TC and TG were measured by assessing the amount of H_2_O_2_ produced (23). For measurement of the HDL-C content, chemical precipitation of lipoproteins containing apoprotein B was performed using dextran sulfate-Mg^2+^. HDL-C was then measured by coupling the product of cholesterol oxidase reaction with an indicator reaction as described (24). The amount of LDL-C was calculated based on the values for TC, TG and HDL-C (25).

### *LXRα* transcript level quantification

To extract RNA, 50 mg of the frozen liver tissue was homogenized. Total RNA was isolated by the RNA-Plus kit (CinnaGen, Iran) according to the manufacturer’s instructions. Next, the extracted RNA solution was decontaminated from any DNA using RNase free DNaseI (Fermentas, Lithuania). Two µg of RNA from each sample was used for synthesizing the first strand cDNA using cDNA synthesis kit (Fermentas, Lithuania) by utilizing oligodT primers. Realtime (SYBRGreen) polymerase chain reaction (PCR) was carried out in a thermal cycler (BioRad, CA, USA) as suggested by the protocol (TaKaRa, Japan). The PCR mixture contained 10 µL Rotor-Gene SYBR Green PCR Master Mix (TaKaRa, Japan), 3 pM of each primer and 25 ng cDNA in a final volume of 20 µL. 

The *LXRα*-specific real-time PCR primers were

F: 5´-CCTGATGTTTCTCCTGACTC-3´

 R: 5´-TGACTCCAACCCTATCCTTA-3´. 

In case of the *Rnβ-actin*

 F: 5´-GGAGAAGATTTGGCACCACAC-3´

 R:5´-GGATGGCTACGTACATGGCTG-3´. 

All primers were purchased from Metabion (Germany). 

Relative mRNA concentrations were calculated from take-off point of reactions (Ct) using the software provided by the manufacturer and normalized to *β-actin* expression level. All measurements were done in duplicates and data were analyzed according to the ∆∆Ct method as our previous study (1). 

### Statistical analysis

All results were expressed as mean ± SEM (standard error of mean) obtained from three independent replicated observations. Changes in all variables were analyzed by independent t test. All statistical analysis was performed by SPSS (Version 13). P<0.01 were considered significant. 

## Results

**Fig.1:**
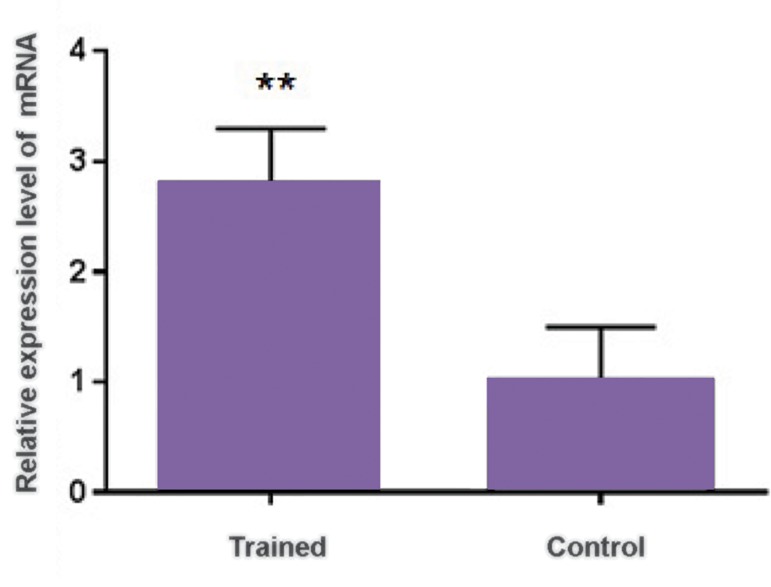
The relative expression level of *LXRα* normalized to *β-actin* gene in trained and control groups. **; Significant difference between trained and control samples at P<0.01.

*LXRα* expression in liver, plasma lipoprotein (HDL-C, LDL-C) and lipid (TC and TG) profiles were examined in rats. *LXRα* expression was significantly increased 2.8 fold (P<0.01) in trained rats (Fig.1). Plasma HDL-C was also significantly higher in trained rats (P<0.01). On the other hand, plasma LDL-C was significantly decreased (P<0.01) in trained rats (Table 1). Plasma TC concentration (P<0.02), and TC/HDL-C (P<0.001) and LDL/HDL-C (P<0.002) ratios significantly decreased in trained rats following the 4-week exercise. However, we observed no significant change in TG concentration (P<0.6) (Table 1).

**Table 1:** Comparison of lipid and lipoprotein profiles, and TC/HDL-C and LDL/HDL-C ratios between control and trained Wistar male rats


Variables	Training group (n=6)	Control group (n=6)	P value

HDL-C (mg/dL)	39.83 ± 1.4	35.16 ± 0.7	0.01
LDL-C (mg/dL)	6.33 ± 0.8	9.83 ± 0.79	0.01
TG (mg/dL)	50.5 ± 2.3	51.83 ± 2.27	0.6
TC (mg/dL)	47.66 ± 2.18	61 ± 4.36	0.02
TC-HDL-C ratio	1.09 ± 0.06	1.72 ± 0. 1	0.001
LDL-HDL-C ratio	0.14 ± 0.01	0.28 ± 0.02	0.002


Data are expressed as mean ± SEM. HDL-C; High-density lipoprotein cholesterol, LDL-C; Low-density lipoprotein cholesterol, TG; Triglyceride, and TC; Total cholesterol.

## Discussion

The major finding of the present study is the significant increase in *LXRα* expression level as a result of performing endurance training. Despite the importance of *LXRα* in cholesterol metabolism, studies have rarely investigated the effect of exercise and exercise training on *LXRα* transcript level. A similar study which analyzed the effect of physical activity on *LXR* reported that low intensity activity (walking 10,000 steps, 3 times a week) resulted in a significant upregulation in *LXR* expression in human leukocytes (20). Baranowski et al. (26) demonstrated that the activation of *LXRα* increased fatty acid utilization during exercise and prevented fatigue caused by glucose insufficiency.

According to Khabazian et al. (27), hepatic *ABCA1* expression was increased in liver after a 6-week exercise on a treadmill with a speed of 26 meters/minute (0° slope) for 60 minutes a day, 5 days a week. Similarly, Ghanbari-Niaki et al. (2) reported increased *ABCA1* expression in rats that had exercised on a treadmill for 5 days per week for 6 weeks with a speed of 25 meters/minute and 90 minutes per session. More recently, Kazeminasab et al. (1) showed that liver *LXRα* and *ABCA1* expression was up-regulated in rats trained for a prolonged period of exercise during 8 weeks. All studies therefore consistently suggest that regular endurance exercise can cause an enhancement in liver *ABCA1* gene expression in rats. 

The higher plasma HDL-C levels observed in this study could be explained by exercise training-induced changes in several parameters of HDL-C metabolism and HDL-C remodeling factors including LCAT, HL, CETP, LPL (28, 29) and ABCA1 (7). Our results are not in line with those by previous studies which reported reduced HDL levels after an exercise activity (29, 30). Although a few studies have shown that exercise activity has no significant effect on HDL levels, the majority of the studies have reported an increase in HDL level after performing exercise activities (31, 32).

We also observed a significant reduction in LDL-C and TC following an exercise session which were in good agreement with previous studies (33, 34) but not all (27, 35).

## Conclusion

We show that treadmill running induces higher plasma HDL-C levels due to the higher ABAC1 expression in liver, a key element in the RCT process which is in turn due to the up-regulation of *LXRα*. Further investigation is required to clarify the effect of different intensities and various modes of exercise on liver *LXRα* expression.
